# The Number of Comorbidities Predicts Renal Outcomes in Patients with Stage 3–5 Chronic Kidney Disease

**DOI:** 10.3390/jcm7120493

**Published:** 2018-11-28

**Authors:** Wen-Chin Lee, Yueh-Ting Lee, Lung-Chih Li, Hwee-Yeong Ng, Wei-Hung Kuo, Pei-Ting Lin, Ying-Chun Liao, Terry Ting-Yu Chiou, Chien-Te Lee

**Affiliations:** Division of Nephrology, Department of Internal Medicine, Kaohsiung Chang Gung Memorial Hospital and Chang Gung University College of Medicine, Kaohsiung 83301, Taiwan; leewenchin@gmail.com (W.-C.L.); yuai@cgmh.org.tw (Y.-T.L.); r5239@cgmh.org.tw (L.-C.L.); stan@cgmh.org.tw (H.-Y.N.); b8701144@cgmh.org.tw (W.-H.K.); r40391132@gmail.com (P.-T.L.); ckd@cgmh.org.tw (Y.-C.L.); tytc107@gmail.com (T.T.-Y.C.)

**Keywords:** chronic kidney disease, multimorbidity, renal outcomes

## Abstract

Background: Chronic kidney disease (CKD) is a global health threat affecting approximately 10% of the adult population worldwide. Multimorbidity is common in CKD, but its impacts on disease outcomes are seldom investigated. Methods: This prospective cohort analysis followed patients, who were part of a multidisciplinary CKD care program, for 10 years. We aimed to determine the impact of multimorbidity on renal outcomes. Results: Overall, 1463 patients with stage 3–5 CKD were enrolled and stratified by the number of comorbidities. Mean follow-up time was 6.39 ± 1.19 years. We found that stage 3–5 CKD patients with at least three comorbidities at enrollment initiated dialysis earlier (hazard ratio (HR): 2.971) than patients without comorbidities. Risk factors for multimorbidity included old age, smoking, and proteinuria. Conclusions: By analyzing the number of comorbidities, a simple and readily applicable method, we demonstrated an association between multimorbidity and poor renal outcomes in stage 3–5 CKD patients. In addition to current guideline-based approaches, our results suggest an urgent need for tailored CKD care strategies for high-risk groups.

## 1. Introduction

Chronic kidney disease (CKD) is an emerging global health threat, affecting an estimated 10% of the adult population worldwide, which is twice the estimated prevalence of diabetes [1,2]. This debilitating disease affects multiple organ systems in the human body and is associated with a markedly increased risk of cardiovascular morbidity and mortality [3,4,5,6,7]. According to the 2016 Global Burden of Disease study, CKD ranked 30th on the list of causes of global deaths in 1990 but rose to 22nd in 2016 [8]. Current strategies for managing CKD include enhancing awareness of CKD among patients, caregivers, and clinicians, guideline-based recommendations to reduce associated complications and slow CKD progression, and the integration of self-management interventions [9,10]. Benefits of these approaches have been reported, but some challenges remain.

Multimorbidity, defined as the presence of two or more chronic health conditions in an individual [11], is a growing concern whilst caring for CKD patients [11,12,13,14]. Some individual comorbidities are known risk factors for CKD progression. In addition, multimorbidity may increase the treatment burden on patients, lead to polypharmacy, and have negative impacts on patient quality of life. The prognostic significance of the number of comorbidities on renal outcomes in CKD patients is not well understood. This study aimed to assess the impact of comorbidities on renal outcomes in patients with stage 3–5 CKD.

## 2. Materials and Methods

### 2.1. Patients

Patients, living in geographically different areas in south Taiwan, were recruited from various outpatient clinics in Kaohsiung Chang Gung Memorial Hospital. They were referred for a multidisciplinary care program between January 1996 and January 2017. Patients included in the study were ≥18 years of age, met the Kidney Disease Outcomes Quality Initiative (KDOQI) criteria for CKD stage 3–5, and were able to give informed consent. Patients who had previously received an organ transplant or had terminal illness were excluded. Participants underwent medical history review, clinical assessment, and blood and urine tests. The 12 comorbidities included in this study were diabetes, hypertension, gout, congestive heart failure, ischemic heart disease, cerebrovascular disease, liver disease, malignancy, tuberculosis, hyperlipidemia, anemia, and connective tissue disease. These comorbidities were pragmatically chosen because they represent a broad spectrum of chronic conditions prevalent among CKD patients and were readily identifiable from patient reports, medication history, and laboratory data. The number of comorbidities was calculated by self-reported history of doctor-diagnosed conditions, disease-specific medications, or laboratory results. Patients were prospectively followed for 10 years, and data were collected until September 2017. This study was approved by the Chang Gung Medical Foundation Institutional Review Board. All methods were performed in accordance with the relevant guidelines and regulations. Informed consent was obtained from all participants.

### 2.2. Statistical Analyses

Descriptive statistics were used to summarize the frequency and distribution of the numbers and types of comorbidities in the study cohort. Based on the number of comorbidities, the burden of comorbidity was categorized into four groups: Zero, one, two, and at least three comorbidities. Univariate and multivariable logistic regression models were used to examine the risk factors for multimorbidity. Survival curves were constructed using the Kaplan–Meier method and evaluated with the log-rank test. Kaplan–Meier plots and multivariate Cox proportional hazards models were used to investigate associations between the number of comorbidities and renal/patient outcomes. The outcome measures used in this study included death and dialysis initiation. The differences in annual estimated glomerular filtration rate (eGFR) decline rate between groups stratified by the number of comorbidities were evaluated by one-way analysis of variance (ANOVA) and the Tukey honestly significant difference (HSD) post hoc test (α = 0.05). Statistical analyses were performed using IBM SPSS Statistics for Windows, Version 19.0. (IBM Corp., Armonk, New York, NY, USA).

## 3. Results

### 3.1. Demographic and Clinical Characteristics of Patients at Referral

We recruited 819 stage 3 CKD patients (mean age 71.5 years; mean baseline eGFR was 37.4 mL/min/1.73 m^2^; male 68%), 468 stage 4 CKD patients (mean age 72.4 years; mean baseline eGFR was 17.6 mL/min/1.73 m^2^; male 55%), and 176 stage 5 CKD patients (mean age 70.8 years; mean baseline eGFR was 8.4 mL/min/1.73 m^2^; male 44%). Mean follow-up time was 6.39 ± 1.19 years. Among the total 1463 patients, 255 (17.4%) had no comorbidities. Of the total patients, 594 (40.6%), 340 (23.2%), and 274 (18.7%) had one, two, and at least three comorbidities, respectively (Table 1). Among all participants, 41.9% met the definition of multimorbidity. Patients aged 65 years or older accounted for 74.2% of the study population.

### 3.2. Prevalence of Comorbidities in Stage 3–5 CKD Patients

The prevalence of individual comorbidities is shown in Table 2. Approximately 41.9% of patients were multimorbid (Table 1). The three most common comorbidities were hypertension, diabetes, and hyperlipidemia. In CKD patients with at least three comorbidities, the prevalence of hypertension approached 90%.

### 3.3. Factors Associated with Multimorbidity

Multivariable logistic regression analysis revealed that age, smoking, and proteinuria were associated with an increased risk of multimorbidity (Table 3). Age above 65 years was associated with a 1.759-fold (95% confidence interval (CI): 1.34–2.30; *p* < 0.001) increased risk of multimorbidity. Current smoking was associated with a higher risk (odds ratio (OR): 1.908) of multimorbidity (95% CI: 1.29–2.81; *p* = 0.001). Those with proteinuria carried a higher risk of multimorbidity (OR = 1.492; 95% CI: 1.20–1.84; *p* < 0.001).

### 3.4. Number of Comorbidities Predicts Poor Renal Outcomes

**Figure 1:**
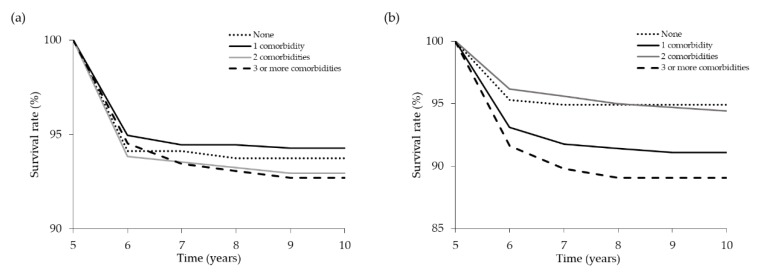
Kaplan–Meier plots showing cumulative patient (**a**) and renal (**b**) survival by comorbidity status.

**Figure 2:**
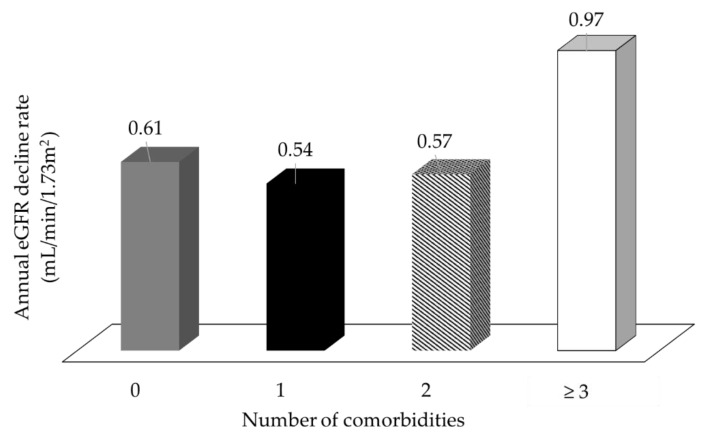
A number of comorbidities ≥3 was associated with a faster decline of the renal function in patients with stage 3–5 CKD.

The Kaplan–Meier method was used to investigate the effect of the number of comorbidities on renal and patient survival. End point for renal survival analysis was the initiation of long-term dialysis. Renal and all-cause patient survival rates at 10 years were determined. The number of comorbidities had no impact on all-cause patient survival at 10 years (Figure 1a). Among 1463 patients, 94 died during the follow-up period. The 10-year patient survival rates were 93.7%, 94.3%, 92.9%, and 92.7% in patients with 0, 1, 2, and ≥3 comorbidities, respectively. However, the Kaplan–Meier plots showed lower renal survival rates (Figure 1b) in patients with a higher number of comorbidities. The 10-year renal survival rates were 94.9%, 91.1%, 94.4%, and 89.1% in patients with 0, 1, 2, and ≥3 comorbidities, respectively. In patients with stage 3–5 CKD, the presence of at least three comorbidities at the time of referral to the multidisciplinary care program was an independent predictor of early dialysis initiation (log rank *p* = 0.014). We also found the annual eGFR decline rates in patients with 0, 1, 2, and ≥3 comorbidities were 0.61, 0.54, 0.57, and 0.97 mL/min/1.73 m^2^, respectively (Figure 2). By ANOVA, we found that patients with at least three comorbidities had faster CKD progression (*p* = 0.022). The differences between study groups were verified by Tukey HSD post hoc test. On univariate Cox regression analyses, patients with three or more comorbidities were associated with an increased risk of dialysis initiation (HR = 2.237; 95% CI: 1.16–4.29; *p* = 0.015). After adjustment for sociodemographic and clinical variables, the relationship between the number of comorbidities and dialysis initiation was accentuated (from 2.237 (95% CI:1.16–4.29) to 2.971 (95% CI:1.53–5.76); *p* = 0.001) for three or more comorbidities compared to no comorbidities (Table 4).

## 4. Discussion

Multimorbidity is common in CKD populations, but a tool to accurately assess its impacts on CKD patients is yet to be determined. The Charlson comorbidity index (CCI) score, originally developed to predict mortality [15], has been used to predict mortality in dialysis patients [16,17,18,19] and renal transplant recipients [20]. However, most of these studies arbitrarily adjusted the weight of individual comorbidities described in the CCI. Another drawback of the CCI score is the inability to reflect the negative impacts of comorbidity on the progressively deteriorating renal function in CKD patients.

Determining the number of comorbidities has recently been reported as a simple, readily applicable, and valid method for examining the impacts of comorbidities on CKD patients [11,12]. A large Canadian database study has highlighted the importance of comorbidities in CKD. By analyzing administrative data from 530,771 CKD patients, Tonelli et al. showed that a higher degree of comorbidity was associated with poorer outcomes, such as all-cause mortality, hospitalization, and increased length of hospital stay [21]. Our data did not show an association between multimorbidity and all-cause mortality. Interestingly, we did show that multimorbidity may be directly associated with early dialysis initiation.

Slowing down the deterioration of renal function is an important task in caring for stage 3–5 CKD patients in whom multimorbidity is common [22,23]. Treating comorbidities in these patients originally aimed to reduce disease burden, thus slowing the deterioration of the renal function. However, current treatment strategies may introduce treatment burden, consisting of polypharmacy, attending multiple appointments, self-management of conditions [24], and psychological distress [25]. This treatment burden may lead to negative outcomes such as non-adherence, reduced quality of life, and wasted resources [26,27,28], which may form a barrier to successful CKD care [26]. Our analysis using multivariable logistic regression identified old age, smoking, and proteinuria as risk factors for multimorbidity in stage 3–5 CKD patients. In studies not specifically designed for CKD populations, old age, smoking, and low educational status have been reported as risk factors for multimorbidity [29,30,31]. Our results are consistent with these studies and demonstrate that these factors are risk factors for multimorbidity in CKD patients. Notably, proteinuria in our analysis is also a potential risk factor for multimorbidity. Proteinuria is not commonly self-reported by patients and requires laboratory tests for diagnosis. Identifying patients with proteinuria not only helped identify kidney damage but also warned of the possibility of multimorbidity in these CKD patients. More importantly, our data demonstrate a link between multimorbidity and worse renal outcomes. As multicomorbidity has been shown to increase both disease and treatment burden in CKD patients [11,26], a tailored CKD care strategy to minimize these burdens may help to improve renal outcomes.

Guideline-based multidisciplinary CKD care has shown great success in treatment goal attainment, preserving impaired renal function, and reducing mortality in CKD patients [32,33,34]. As multimorbidity is becoming more prevalent in CKD populations, lessons from managing multimorbidity in general populations are valuable [35]. These include attempts to minimize patients’ burden, increase patients’ capacity to cope with these burdens, and integrate disease-specific guidelines [11,35]. Although we offered guideline-based multidisciplinary CKD care to our patients, we uncovered that multimorbidity significantly and negatively affected renal outcomes. The implication of our findings is that clinicians need to integrate patients’ age and habits (e.g., smoking), the presence of proteinuria, the number of comorbidities, and the disease-specific guidelines to build a comprehensive care plan for individual CKD patients. 

## 5. Conclusions

The determination of the number of comorbidities in patients provides a simple, readily applicable, and valid method for classifying comorbidities and predicting renal outcomes in patients with stage 3–5 CKD. The number of comorbidities present should be considered when making tailored patient care plans.

## Figures and Tables

**Table 1:** Demographic and clinical characteristics of the patients at referral.

		CKD Stage			
Characteristic	Category	3	4	5	Total *n* = 1463
		Total *n* = 819	Total *n* = 468	Total *n* = 176	
		*n* (%)	*n* (%)	*n* (%)	*n* (% of total)
Sex	Male	555 (67.8)	256 (54.7)	78 (44.3)	889 (60.8)
	Female	264 (32.2)	212 (45.3)	98 (55.7)	574 (39.2)
Age	mean(SD)	71.5 (12.3)	72.4 (12.2)	70.8 (12.5)	71.7 (12.3)
	18–64	207 (25.3)	117 (25.0)	54 (30.7)	378 (25.8)
	65+	612 (74.7)	351 (75.0)	122 (69.3)	1085 (74.2)
Education status	No formal or elementary school	407 (49.7)	279 (59.6)	110 (62.5)	796 (54.4)
	High school	280 (34.2)	141 (30.1)	55 (31.3)	476 (32.5)
	College or higher	132 (16.1)	48 (10.3)	11 (6.3)	191 (13.1)
Smoking	Current	94 (11.5)	41 (8.8)	6 (3.4)	141 (9.6)
	Ex-smoker	183 (22.3)	85 (18.2)	27 (15.3)	295 (20.2)
	Never	542 (66.2)	342 (73.1)	143 (81.3)	1027 (70.2)
Proteinuria	Current	353 (43.1)	202 (43.2)	91 (51.7)	646 (44.2)
	No proteinuria	466 (56.9)	266 (56.8)	85 (48.3)	817 (55.8)
Number of comorbidities	None	145 (17.7)	79 (16.9)	31 (17.6)	255 (17.4)
	1	332 (40.5)	190 (40.6)	72 (40.9)	594 (40.6)
	2	194 (23.7)	104 (22.2)	42 (23.9)	340 (23.2)
	3 or more	148 (18.1)	95 (20.3)	31 (17.6)	274 (18.7)

CKD, Chronic kidney disease; SD, standard deviation.

**Table 2:** Prevalence of individual comorbidities at baseline.

Comorbidity	CKD Stage 3 Total *n* = 819		CKD Stage 4 Total *n* = 468		CKD Stage 5 Total *n* = 176		Total	
	*n*	Prevalence (%)	*n*	Prevalence (%)	*n*	Prevalence (%)	*n*	Prevalence (%)
Hypertension	547	66.8	307	65.6	117	66.5	971	66.4
Diabetes	265	32.4	164	35.0	57	32.4	486	33.2
Hyperlipidemia	101	12.3	52	11.1	14	8.0	167	11.4
Cerebrovascular disease	59	7.2	24	5.1	8	4.5	91	6.2
Malignancy	33	4.0	23	4.9	5	2.8	61	4.2
Liver disease	32	3.9	9	1.9	5	2.8	46	3.1
Anaemia	8	1.0	14	3.0	11	6.3	33	2.3
Ischemic heart disease	13	1.6	12	2.6	3	1.7	28	1.9
Gout	13	1.6	12	2.6	3	1.7	28	1.9
Connective tissue disease	6	0.7	4	0.9	2	1.1	12	0.8
Congestive heart failure	3	0.4	5	1.1	1	0.6	9	0.6
Tuberculosis	1	0.1	2	0.2	1	0.6	4	0.3

**Table 3:** Factors associated with multimorbidity.

Variable	Two or More Comorbidities (vs. One or Fewer)				
		Univariate		Multivariable *	
		OR (95 % CI)	*p*-Value	OR (95 % CI)	*p*-Value
Age (vs. 65+)		1.560 (1.22–1.99)	<0.001	1.759 (1.34–2.30)	<0.001
Sex (male vs. female)		0.944 (0.76–1.16)	0.595	1.152 (0.88–1.49)	0.289
Education status (vs. no formal or elementary school)	High school	0.967 (0.76–1.21)	0.774	1.089 (0.84–1.40)	0.509
	College or higher	0.825 (0.59–1.14)	0.246	1.018 (0.71–1.45)	0.923
Smoking (vs. non-smokers)	Current smoker	1.616 (1.13–2.30)	0.008	1.908 (1.29–2.81)	0.001
	Ex-smoker	1.202 (0.92–1.56)	0.168	1.270 (0.94–1.71)	0.118
eGFR at study entry (mL/min per 1.73 m^2^, continuous)		0.999 (0.99–1.00)	0.798	0.998 (0.99–1.00)	0.639
Proteinuria (vs. no proteinuria)		1.405 (1.14–1.73)	0.001	1.492 (1.20–1.84)	<0.001

* Adjusted for old age, sex, education status, smoking, estimated glomerular filtration rate (eGFR), and proteinuria; OR, odds ratio; CI, confidence interval.

**Table 4:** Cox proportional hazards models for dialysis initiation in the study population.

Variable		Model 1 (Univariate)		Model 2 (Sociodemographic and Clinical Variables)	
		HR (95 % CI)	*p*-Value	HR (95 % CI)	*p*-Value
Number of comorbidities (vs none)	1	1.708 (0.93–3.13)	0.084	2.032 (1.10–3.74)	0.023
	2	1.023 (0.50–2.07)	0.950	1.347 (0.65–2.75)	0.415
	3 or more	2.237 (1.16–4.29)	0.015	2.971 (1.53–5.76)	0.001
Age (vs. 65+)				0.463 (0.30–0.69)	<0.001
Sex (male vs. female)				0.730 (0.46–1.13)	0.162
Education status (vs. no formal or low level of qualifications)	High school			1.269 (0.83–1.93)	0.266
	College or higher			0.683 (0.34–1.36)	0.282
Smoking (vs. non-smokers)	Current smoker			0.970 (0.47–1.97)	0.933
	Ex-smoker			1.049 (0.61–1.79)	0.863
eGFR at study entry (mL/min per 1.73 m^2^, continuous)				0.932 (0.91–0.94)	<0.001
Proteinuria (vs. no proteinuria)				1.286 (0.88–1.87)	0.191

Outcome—Dialysis initiation, n events = 115; Model 1: univariate; Model 2: adjusted for old age, sex, education status, smoking, eGFR, and proteinuria.
